# Case report: Dissolving carotid plaque associated to Lorlatinib-related dyslipidemia

**DOI:** 10.3389/fonc.2024.1322501

**Published:** 2024-03-05

**Authors:** Lukas Mayer-Suess, Michael Knoflach, Andreas Pircher, Stefan Kiechl, Christoph Schmidauer, Eva Hametner

**Affiliations:** ^1^ Department of Neurology, Medical University of Innsbruck, Innsbruck, Austria; ^2^ VASCage, Centre on Clinical Stroke Research, Innsbruck, Austria; ^3^ Department of Internal Medicine V, Hematology and Oncology, Medical University of Innsbruck, Innsbruck, Austria

**Keywords:** Lorlatinib, LDL-C, dyslipidemia, stroke, case report

## Abstract

We present a case with prolonged Lorlatinib-related dyslipidemia causing internal carotid artery stenosis, putting the patient at risk of cerebrovascular events. Through intensified lipid-lowering treatment and dose reduction of Lorlatinib, LDL-C levels decreased markedly. Surprisingly, the left sided internal carotid artery stenosis dissolved accordingly. Due to the high efficacy of the new selective tyrosine kinase inhibitors and resulting long-term treatment, it is essential to carefully follow-up and include drug specific side effect monitoring. This case emphasizes that Loraltinib-related dyslipidemia has to be taken seriously and treatment should be initiated as promptly as possible. We conclude that in cases were lipid dysregulation remains and Lorlatinib treatment has to be continued, cerebrovascular appraisal through ultrasound should be considered and, if stenosis is evident, intensified treatment regimen of dyslipidemia or dose reduction of Lorlatinib should be discussed in an interdisciplinary setting.

## Introduction

Lorlatinib, a third-generation anaplastic lymphoma kinase (ALK) and c-ros oncogene 1 (ROS1) tyrosine kinase inhibitor, is an established safe and effective therapy for patients with ALK rearranged non-small cell lung cancer (NSCLC) (1–3). Recently the pivotal phase III Crown trial showed that Lorlatinib is an effective first line ALK inhibitor treatment in individuals with NSCLC (4). One of the most frequent adverse effects of Lorlatinib-treatment is dyslipidemia with about 80% and 60% of treated individuals having hypercholesterol- or hypertriglycidemia respectively (5). It is common knowledge that prolonged dyslipidemia in general increases the risk of macroangiopathy and ischemic stroke on the long-run. Whether Lorlatinib-related dyslipidemia has any significance in that regard in the medium term is unknown (6). We present the case of a female NSCLC patient with marked Lorlatinib-related, primarily LDL-C driven, dyslipidemia and emphasize on the importance of timely treatment of adverse drug effects to enable continued targeted therapies in these patients.

## Case presentation, diagnostic work up and therapeutic interventions

**Figure 1:**
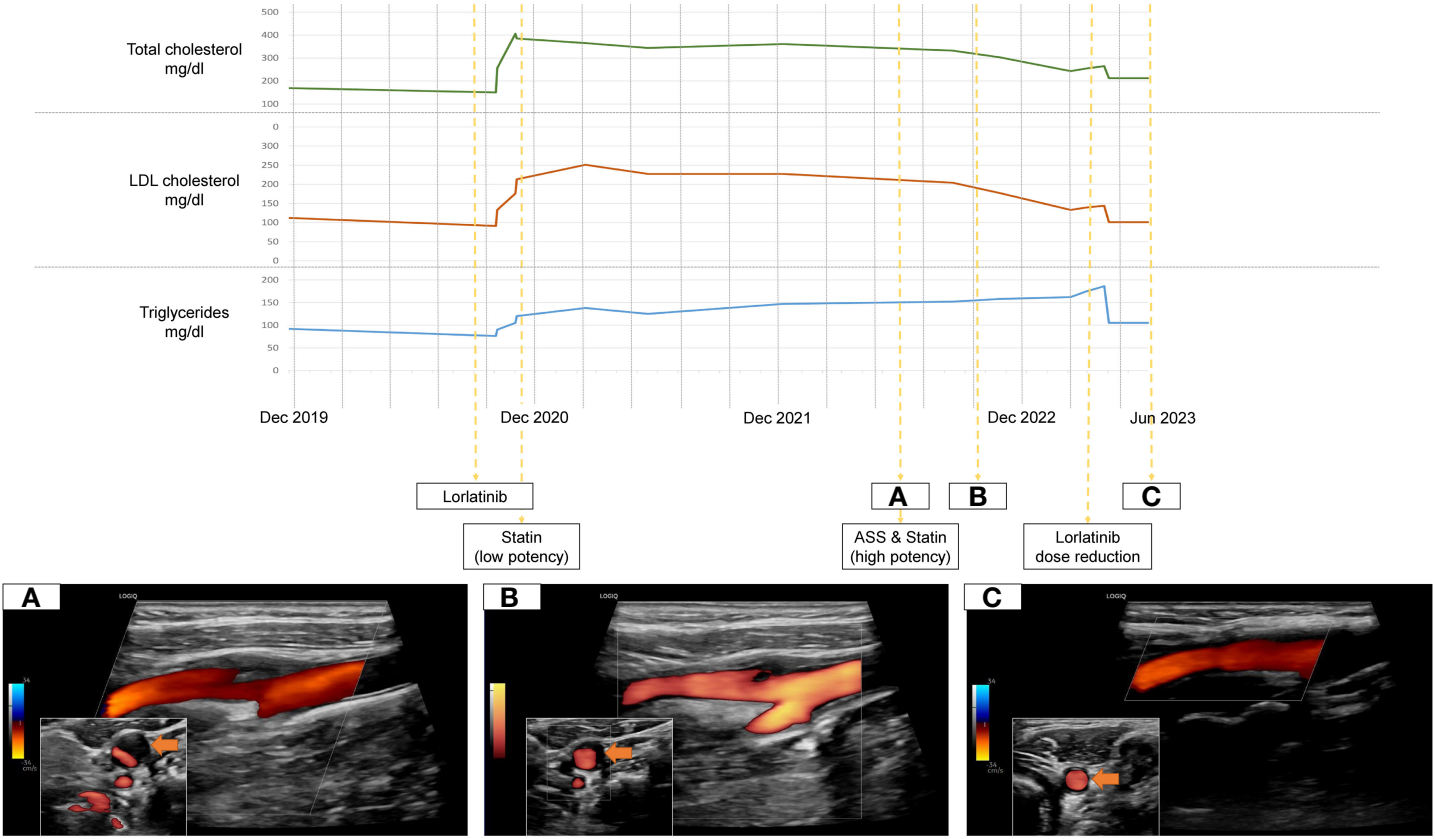
Timeline of treatment, Lorlatinib-related dyslipidemia and dissolving carotid plaque under lipid-lowering therapy **(A-C)**.

We present a 53 year old female patient who began experiencing exertional dyspnoea in August of 2019. Her prior medical history as well as annual routine health examinations, which included carotid ultrasound, were uneventful. The patient had no relevant family history to report. **Figure 1** holds information on relevant data from the episode of care, specific to our case report.Within a month after symptom onset, dyspnoea worsened so that the patient was unable to lie flat on her back. Therefore, the patient sought for medical attention. The initial chest X-ray revealed significant right sided pleural effusion. In follow-up imaging, computed tomography showed a central mass in the posterobasal segment of the right lung, which, in addition to multiple smaller intrapulmonary lesions, had marked increase in glucose metabolism in a positron emission tomography. Integrating histopathology and molecular markers into the diagnosis, the patient was diagnosed with a stage III ALK positive adenocarcinoma of the lung. Dyspnoea resolved after drainage of the pleural effusion and treatment with Alectinib, a second generation ALK tyrosine kinase inhibitor (600mg twice daily), was promptly initiated after multidisciplinary discussion in our in-house tumor board from December onward. Within a year, she developed secondary ALK resistance mutation G1202R, which led to a second line treatment with the third-generation ALK tyrosine kinase inhibitor Lorlatinib (100mg/day - initiation in October of 2020). Subsequently, the patient had partial remission of NSCLC but developed an LDL-C driven dyslipidemia in need of lipid-lowering therapy (**Figure 1**). The treating oncologists chose Pravastatin, a low potency statin. In August of 2022 a routine health examination, which included carotid ultrasound, revealed a previously unknown asymptomatic left sided carotid artery plaque causing relevant stenosis which was confirmed in our neurovascular outpatient clinic (**Figure 1A**). Due to this finding, the lipid-lowering treatment was modified from low- to a high-potency statin (Pravastatin 40mg per day to Rosuvastatin 40mg per day) and an antiplatelet agent was added in an effort of primary stroke prevention. In March of 2023 the patient showed NSCLC disease progression despite Lorlatinib attributable to further ALK resistency mutations (p.IIe1171Asn and p.Asp1203Asn). Therefore, treatment strategies were adapted with Lorlatinib dose being reduced to 50mg per day from March 2023 onward.

## Follow-up and outcomes

Over time, the level of dyslipidemia decreased through the treatment switch from low potency statin to high potency statin treatment but especially due to the dose reduction of Lorlatinib. Surprisingly, the left-sided internal carotid artery plaque dissolved accordingly. (**Figures 1B, C**).

## Discussion

Lung cancer in general is one of the most common cancer types worldwide with NSCLC being the most frequent subtype (7). In 3-7% of NSCLC, ALK, a gene involved in the production of potent oncogenic drivers, is positive (8, 9). In these cases, tyrosine kinase inhibitors were proven to be effective treatment options enabling a substantial increase in patient survival rate (9). Accordingly, Lorlatinib has recently been shown to be such an effective first line ALK inhibitor treatment option in individuals with NSCLC (4). However, treatment may be hindered through issues specific to tyrosine kinase inhibitors, namely the emergence of drug resistance mutations, or due to adverse effects of patients to targeted therapies (8, 10).

We present a case of marked dyslipidemia (i.e. 2.5 fold increase of LDL-C) due to Loraltinib, which is the most common adverse effect of the third-generation ALK/ROS1 tyrosine kinase inhibitor (1, 5). Lorlatinib-related dyslipidemia typically occurs in up to 80% of patients within the initial weeks after treatment initiation. To date, expert recommendations on the clinical management of these cases do not support a dose delay or -reduction of Lorlatinib as it was believed to be an easily manageable (i.e. through lipid-lowering agents) and asymptomatic effect (5).

In our case however, the patient developed a relevant extracranial carotid artery plaque formation causing stenosis, which was attributable to the prolonged Lorlatinib-related lipid dysregulation. Fortunately, the plaque formation responded to adequate treatment adaptation (**Figure 1**). According to current cerebrovascular guidelines, due to the internal carotid artery stenosis, the patient qualified for a very-high risk profile of suffering cerebrovascular events (11). This results in a recommended treatment regimen achieving LDL-C reduction from baseline of ≥50% overall and a target goal of LDL-C <55mg/dL (11). However, Lorlatinib is contraindicated in patients taking CYP3A inducers as it is both a substrate and inducer of the CYP3A itself, severely limiting the choice of statins (12). Current consensus recommendations concerning Lorlatinib-related dyslipidemia can solely attest to one high-potency statin, Rosuvastatin, due to its low involvement with CYP450 enzymes (12). High-intensity statins can potentially reduce LDL-C levels by ≥50% (13). As our patients LDL-C levels were consistently above 200mg/dL and considerable inter-individual variations in effectiveness of statin therapies exist, achieving recommended LDL-C levels <55mg/dL under Rosuvastatin is unrealistic (13). Overall, due to the high efficacy of the new selective tyrosine kinase inhibitors and resulting long term treatment it is essential to carefully follow-up and include drug specific side effect monitoring. Concerning outcome, the last out-patient visit of our case was in January of 2024. Due to continued dose reduction of Lorlatinib and high intensity statin treatment, the betterment of treatment related dyslipidemia remained. Even though statin treatment is associated to plaque volume reduction, mainly in studies of patients with coronary heart disease, a complete resolution of internal carotid plaques is unique and cannot solely be attributed to statin initiation (14).

A limitation of our case is the missing ultrasound image file before Lorlatinib-treatment. Further, it would have been rewarding to apply image analysis tools to better compare sequential ultrasound examinations (15).

This case emphasizes that Loraltinib-related dyslipidemia has to be taken seriously and treatment should be initiated as promptly as possible (6). In cases were lipid dysregulation remains and Lorlatinib-treatment has to be continued, cerebrovascular appraisal through ultrasound should be considered and, if stenosis is evident, intensified treatment regimen of dyslipidemia, namely PCSK9 inhibitors, or dose reduction of Lorlatinib should be discussed in an interdisciplinary setting.

## Data availability statement

The original contributions presented in the study are included in the article/supplementary material. Further inquiries can be directed to the corresponding author.

## Ethics statement

The studies involving humans were approved by Ethics committee of the Medical University of Innsbruck. The studies were conducted in accordance with the local legislation and institutional requirements. The participants provided their written informed consent to participate in this study. Written informed consent was obtained from the individual(s) for the publication of any potentially identifiable images or data included in this article.

## Author contributions

LM-S: Conceptualization, Data curation, Formal analysis, Investigation, Methodology, Project administration, Visualization, Writing – original draft. MK: Supervision, Validation, Writing – review & editing. AP: Supervision, Validation, Writing – review & editing. SK: Resources, Supervision, Writing – review & editing. CS: Resources, Supervision, Writing – review & editing. EH: Resources, Supervision, Writing – review & editing.
